# Nom1 Mediates Pancreas Development by Regulating Ribosome Biogenesis in Zebrafish

**DOI:** 10.1371/journal.pone.0100796

**Published:** 2014-06-26

**Authors:** Wei Qin, Zelin Chen, Yihan Zhang, Ruibin Yan, Guanrong Yan, Song Li, Hanbing Zhong, Shuo Lin

**Affiliations:** 1 Laboratory of Chemical Genomics, School of Chemical Biology and Biotechnology, Peking University Shenzhen Graduate School, Shenzhen, China; 2 Department of Biology, South University of Science and Technology of China, Shenzhen, Guangdong, China; 3 Department of Molecular, Cell and Developmental Biology, University of California, Los Angeles, Los Angeles, California, United States of America; University of Louisville, United States of America

## Abstract

Ribosome biogenesis is an important biological process for proper cellular function and development. Defects leading to improper ribosome biogenesis can cause diseases such as Diamond-Blackfan anemia and Shwachman-Bodian-Diamond syndrome. Nucleolar proteins are a large family of proteins and are involved in many cellular processes, including the regulation of ribosome biogenesis. Through a forward genetic screen and positional cloning, we identified and characterized a zebrafish line carrying mutation in *nucleolar protein with MIF4G domain 1 (nom1)*, which encodes a conserved nulceolar protein with a role in pre-rRNA processing. Zebrafish *nom1* mutants exhibit major defects in endoderm development, especially in exocrine pancreas. Further studies revealed that impaired proliferation of *ptf1a*-expressing pancreatic progenitor cells mainly contributed to the phenotype. RNA-seq and molecular analysis showed that ribosome biogenesis and pre-mRNA splicing were both affected in the mutant embryos. Several defects of ribosome assembly have been shown to have a p53-dependent mechanism. In the *nom1* mutant, loss of p53 did not rescue the pancreatic defect, suggesting a p53-independent role. Further studies indicate that protein phosphatase 1 alpha, an interacting protein to Nom1, could partially rescue the pancreatic defect in *nom1* morphants if a human nucleolar localization signal sequence was artificially added. This suggests that targeting Pp1α into the nucleolus by Nom1 is important for pancreatic proliferation. Altogether, our studies revealed a new mechanism involving Nom1 in controlling vertebrate exocrine pancreas formation.

## Introduction

The nucleolus, a non-membrane bound structure within the nucleus of eukaryotic cells, regulates many biological processes, including cell-cycle progression, response to stress, mitosis [1]. Furthermore, one of its major roles is to regulate ribosome biogenesis. Ribosome biogenesis is a tightly controlled process, involving multiple steps to produce and coordinate the assembly of rRNAs, over 80 ribosomal proteins (RPs), approximately 170 associated proteins, as well as many small nucleolar RNAs (snoRNAs) [2]. Any disruption of these components or steps in the assembly of a functional ribosome may affect cell survival and function. Malfunction of nucleolar proteins can thus lead to disruption of the formation of a functional ribosome, and defects in ribosome biogenesis have also been implicated in human diseases such as Dyskeratosis congenita syndrome [3], Werner syndrome [4] and Rothmund-Thomson syndrome [5].

NOM1 is a nucleolar protein firstly identified from the bone marrow of a pediatric patient with acute myeloid leukemia (AML) carrying a translocation between chromosome 12p13 and 7q36 [6]. Its implication in leukemia is unclear since differences in *NOM1* expression level between translocation-positive and -negative AML were not found [7]. The human NOM1 protein has 860 amino acids, and contains an MIF4G domain as well as an MA3 domain. Proteins with MIF4G and/or MA3 domains have been shown to be important in regulating cell growth, proliferation, protein translation, cell transformation, and apoptosis [8]. Nom1 proteins are highly conserved in various species, from yeast to humans. Orthologs of Nom1 have a characteristic nucleolar localization signal sequence (NoLS), MIF4G domains, and MA3 domains. Within the nucleolus, NOM1 co-localizes with B23, a well-known nucleolar protein [9]. Yeast two-hybrid and co-immunoprecipitation (Co-IP) experiments indicate that there is a direct physical interaction between NOM1 proteins and EIF4A1, EIF4A2, EIF4A3, and PP1 [10], [11]. Mutation in Sgd1p, the yeast homolog of human NOM1, results in defects in cell growth and pre-rRNA processing. Meanwhile, siRNA-mediated knockdown of *NOM1* expression in HEK293T cells decreased the rate of 18S rRNA formation [10]. To date, the function of NOM1 in vertebrate development has not been well-studied.

Through a forward genetic screen, we identified a zebrafish mutant (*dg5* mutant), which has major defects in endoderm development, especially in exocrine pancreas. Positional cloning and molecular biology studies revealed that the mutant phenotype was caused by a 5-bp deletion in the coding sequence of the *nom1* gene, resulting in a truncated Nom1 protein. The proliferation, but not the specification of endoderm, was affected in Nom1 deficient embryos. Whole transcriptome analysis by using RNA-seq indicated that ribosome biogenesis and pre-mRNA splicing were affected in the mutant. The pancreatic defect induced by *nom1* mutation was independent of p53 activation, as loss of p53 did not rescue the phenotype. Similar to yeast and cultured human cells, Nom1 deficiency caused a reduction of 18s-RNA formation in zebrafish. Pp1α, an interacting protein to Nom1, when linked to a human NoLS sequence, can partially rescue the pancreas defect in *nom1* morphant. Overall, we report a novel developmental role for the nucleolar protein Nom1.

## Results

### *Dg5* zebrafish mutants have defects in pancreas, intestine, liver and craniofacial development

**Figure 1:**
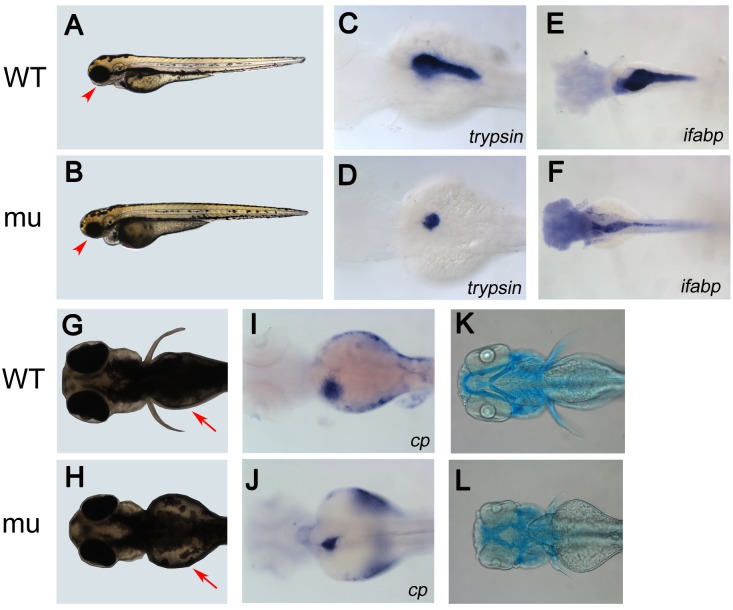
*dg5* mutant has an endodermal and craniofacial defect. (A, B) Lateral and (G, H) dorsal views of live Wild Type (WT) sibling and *dg5* larvae at 3 dpf. Smaller head and eyes can be seen (arrowhead in A, B). Impaired yolk absorption is apparent in *dg5* mutant at 3 dpf (arrows in G, H). (C–F, I–L) All dorsal views, anterior to the left. At 3 dpf, the expression of *trypsin (try)* is markedly reduced in *dg5* mutant (D) as compared to WT group (C). The intestine marker *fatty acid binding protein 2* (*ifabp*) expression reveals that the intestine is thinner in *dg5* larvae (E) as compared to WT group (F) at 3.5 dpf. The expression of *ceruloplasmin* (*cp)* shows that the liver is smaller in *dg5* mutant (H) as compared to WT group (I) at 3 dpf. As compared to control group (K), craniofacial development is abnormal in *dg5* larvae (L) by alcian blue staining at 4 dpf.

Zebrafish *dg5* was isolated as a recessive lethal mutation while screening for *Tol2:GFP* transposon-mediated enhancer trap transgenic lines [12]. Prior to 2.5 days post-fertilization (dpf), no morphological difference was observed between *dg5* mutant embryos and wild-type (WT) siblings; however, starting at 3 dpf, *dg5* mutants exhibited smaller head and eyes as well as impaired yolk absorption (Figure1A, 1B, 1G and 1H). At 7 dpf, *dg5* mutants began to develop severe edema (Figure S4 in File S1) and died by 10 dpf. To determine which organs are affected in *dg5* mutants, we performed RNA whole-mount *in-situ* hybridization (WISH) using various tissue specific gene expression markers. The analysis showed that endoderm markers were predominantly affected in *dg5* mutants, as indicated by expression of *fatty acid binding protein 2 (ifabp), ceruloplasmin* (*cp)*, and *trypsin (try)* (Figure 1C–F and Figure 1 I–J). However, the endocrine markers *insulin* (*ins*), *glucagon a (glu*) and *somatostatin 2 (sst)* were not affected in *dg5* mutant (Figure S1 in File S1). Other markers such as *hbae1* and *mpo* were similar between mutant and WT (Figure S2 in File S1). In addition to defective digestive organs, the *dg5* mutants also exhibited malformation in skeletal development as shown by Alcian Blue staining (Figure1K and 1L). Altogether, these data demonstrate that *dg5* mutants have a specific effect on endoderm development. Using “linker”-mediated PCR (or ligation-mediated PCR) technique [13], we attempted to identify whether there is a gene containing a *tol2* transposon insertion and also linked to *dg5* locus, but failed. Further breeding did not reveal any cosegregation between the mutant phenotype and specific *Tol2* GFP expression, thus we concluded that the phenotype was caused by a spontaneous mutation and, therefore, used a traditional positional cloning approach to identify the gene responsible for the defects.

### *Dg5* locus encodes zebrafish *nom1*

**Figure 2:**
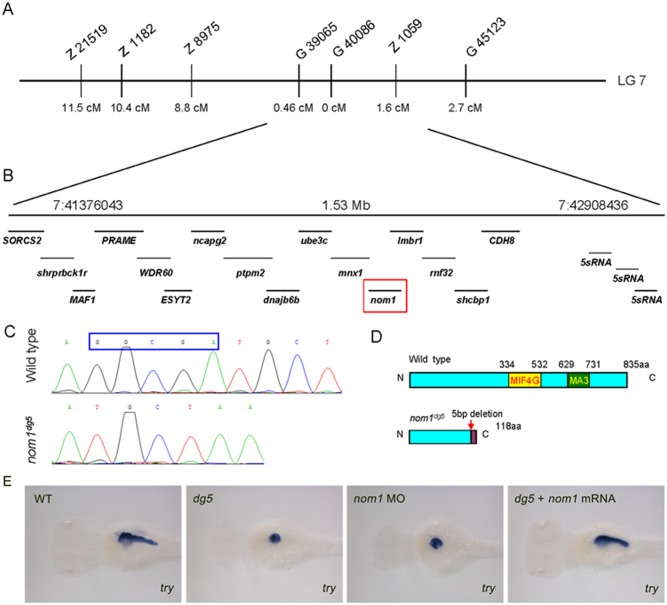
*dg5* encodes zebrafish *nom1*. (A) Chromosome 7 is linked to the *dg5* locus, the cM distance of different SSLP markers to *dg5* locus is marked. (B) The region between markers G39065 and Z1059, contains 16 annotated genes (B). (C) Sequencing shows that *nom1* from *dg5* mutant has a 5-bp nucleotide deletion, producing a truncated *nom1* protein. (E) *nom1* ATG-MO injected embryos show the same decreased *try* expression level as compared to *dg5* mutant and zebrafish *nom1* mRNA can rescue *try* expression. Dorsal views, anterior to the left.

Using bulk segregation analysis, several SSLP (simple sequence length polymorphism) markers on zebrafish chromosome 7 were shown to be linked to the *dg5* locus (Figure 2A). After analyzing the recombination frequencies of Z21519, Z1182, Z8975, G39065, Z1059 and G45123 markers, we found that *dg5* locus was closely linked to the marker G39065 (0.47 cM, 4 recombination events in 854 meioses) and marker Z1059 (1.64 cM, 14 recombination events in 854 meioses). This analysis of 854 mutant larvae positioned the *dg5* locus into a 1530-kilobase interval encompassing 16 annotated genes. Then we identified a zero recombinant marker G40008 in this region (Figure 2A and 2B). By sequencing the open reading frame of genes adjacent to this marker, a 5 base pair deletion was identified in coding region sequence of the zebrafish *nom1* gene. Zebrafish *nom1* encodes a predicted 835 amino-acid protein, which contains the characteristic MIF4G and MA3 domains. It has a 75% similarity compared to human NOM1 protein. The identified mutation is predicted to produce a 118 amino acid truncated Nom1 protein lacking the characteristic MIF4G and MA3 domains. (Figure 2C and 2D).

To confirm that *nom1* is the mutated gene in the *dg5* locus; we utilized antisense morpholinos to knockdown the expression levels of Nom1. Two antisense morpholinos (MO) targeting *nom1* were designed, one targeting the *nom1*’s ATG start codon to block translation and another targeting exon4 splicing site to affect pre-mRNA splicing. RT-PCR revealed that the splice-MO is functional, but at a low efficiency. To validate the ATG-MO efficiency, an EGFP fusion protein with a *nom1* ATG-MO target site was constructed. Injection 150 pg of *EGFP* mRNA contained *nom1* ATG-MO target site into WT embryos resulted in a strong green fluorescence at 5 s stage, and the fluorescence disappeared when 2 ng *nom1* ATG-MO co-injected (Figure S3 in File S1). Because *nom1* ATG-MO was more efficient than the splice-MO, ATG-MO was used in the following validation experiments. Shown by WISH, MO-injection decreased embryonic *try* expression as observed in *dg5* mutants (Figure 2E). Moreover, injecting 200 pg of wild-type *nom1* rescued the *dg5* mutant morphological phenotype (Figure S4 in File S1) and restored *try* expression (Figure 2E). Together, these data support *nom1* as the gene responsible for the *dg5* mutant (*dg5^nom1^*) phenotype.

### *Nom1* is predominantly expressed in the zebrafish digestive organs and brain

**Figure 3:**
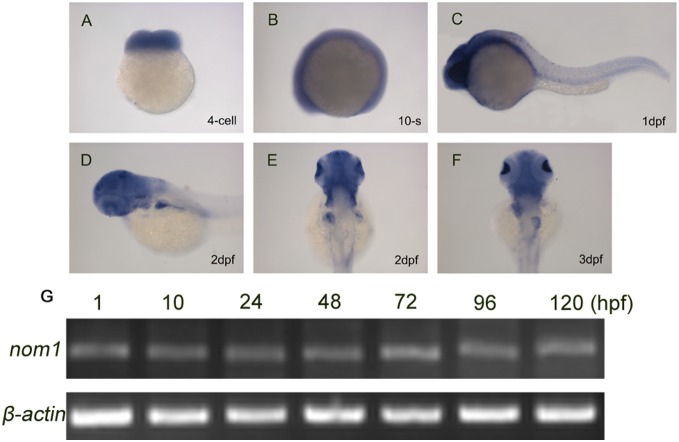
*Nom1* expression in the developing zebrafish. (A–D) Lateral views, anterior to the left. (E, F) Dorsal views, anterior to the top. (A) *nom1* has a strong maternal expression at 4-cell stage. (B–C) Non-specific ubiquitous *nom1* expression is evident at 10-somite stage and 1 dpf. (D, E) At 2 dpf, *nom1* expression in brain and liver is more apparent. (F) In addition to brain, *nom1* is strongly expressed in pancreas and weakly in intestine at 3 dpf. (G) RT-PCR analysis shows that *nom1* transcript is present at all embryonic stages.

To assess the spatiotemporal expression pattern of *nom1* during zebrafish development, we performed WISH and RT-PCR analysis. *Nom1* was maternally expressed during early embryogenesis (Figure 3A, B) and continued to be expressed ubiquitously until 1 dpf (Figure 3C). Then *nom1* expression became restricted to the eye, head, liver and pectoral fin bud by 2 dpf (Figure 3D, E). At 3 dpf, *nom1* expression was detected in the eye, head, liver, pancreas and intestine (Figure 3F). RT-PCR showed that *nom1* had a fairly constant expression from 1 hpf to 5 dpf (Figure 3G). Overall, *nom1* expression pattern is consistent with defects observed in the head, eye and digestive organs in *dg5^nom1^* mutant larvae.

### The pancreas proliferation is affected in *dg5^nom1^* mutant

**Figure 4:**
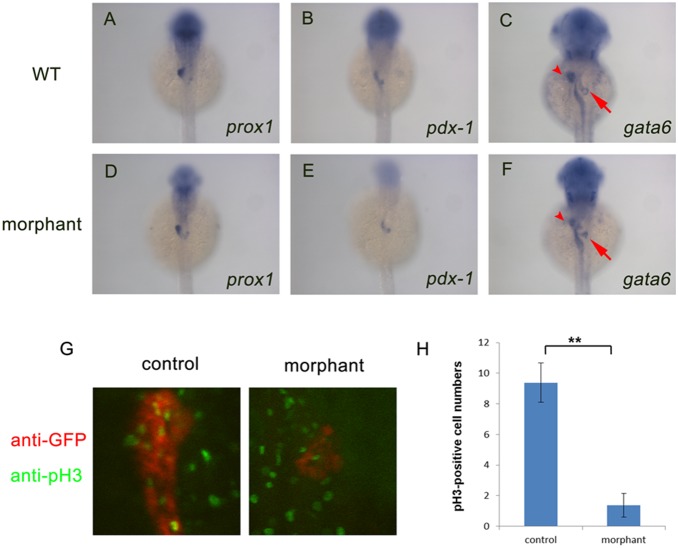
*Nom1* is indispensable for pancreas proliferation but not for specification. (A–F) Dorsal views, anterior to the top. (A, B) Expression of *prox1* (D, E) and *pdx-1* (C, F) in the *nom1* morphants is comparable to that in WT embryos at 36 hpf but reduced *gata6* expression in liver (arrow) and pancreas (arrowhead) of *nom1* morphants is obvious. (G) Anti-phospho Histone H3 (pH 3) staining for control and *nom1* morphant embryos at 3 dpf. Red: GFP staining. Green: pH 3 staining. The signal of pH 3 staining in the pancreas of morphants is recognizably decreased. (H) Quantification of pH 3-positive cell numbers. Data were collected from 10 embryos. Error bars mean±SD. **P<0.01.

Since the exocrine pancreas was the most severely affected, we focused our analysis on *nom1* function during pancreas development. To determine which developmental process was affected by *nom1* mutation, we examined expression of several early endoderm markers at different stages. Expression of *prox1* and *pdx-1,* two of the earliest pancreas specific markers, was not affected in *nom1* MO group at 36 hpf (Figure 4A, B, D, E). However, expression of *gata6,* a pan-endodermal marker, was reduced at 2 dpf (Figure 4 C, F), suggesting pancreas development is affected starting at 2 dpf. Using the *ptf1a:GFP* transgenic line to visualize pancreatic cells, we evaluated the effects of *nom1* MO injection on the developing pancreas. We examined the size of the pancrease at various time points and found that *ptf1a:GFP* expressing pancreatic cells were reduced from 2 dpf and significantly decreased by 3 dpf (Figure S5 in File S1). These findings suggest that *nom1* is dispensable for pancreatic specification but necessary for expansion. To further investigate the mechanism of the hypoplastic pancreas phenotype in *dg5^nom1^* mutants, the proliferation rate of *ptf1a*-expressing pancreatic cells were analyzed by phosphorylated histone H3 (pH 3) staining. As shown in Fig. 4G and 4H, proliferation was decreased dramatically in *nom1* morphants compared to the control group. Meanwhile, TUNEL assay was used to assess apoptosis, and revealed no significant change between the two groups (data not shown). These observations indicate that loss of *nom1* in zebrafish results in a defective proliferation of exocrine pancreatic cells.

### Ribosome-related gene expression is affected in *dg5^nom1^* mutant

**Figure 5:**
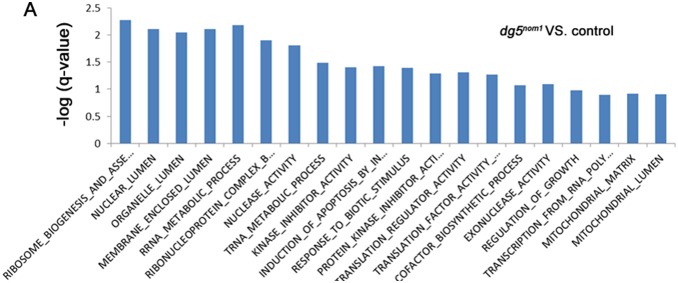
Enrichment analysis of differentially expressed genes in *dg5^nom1^* mutant. The mostly enriched GO categories for genes upregulated in *dg5^nom1^* embryos as compared to control embryos. GO categories are ranked by their *q*-value.

To further understand the consequence of *nom1* deficiency in the developing zebrafish embryos, we performed RNA-Seq (Illumina, HiSeq 2000) analysis of WT and mutant embryos at 2.5 dpf, when mutant embryos could first be identified morphologically. The sequencing result was mapped onto the zebrafish genome, using q-value cutoff of 0.05 to identify genes with significant different expression level [14]. This analysis identified 2419 up-regulated and 1662 down-regulated genes from a total of 48,140 mapped zebrafish genes (Table S1 in File S1). Gene Ontology analysis for functional group categories revealed that these transcripts were enriched in genes associated with ‘ribosome biogenesis’, ‘nuclear lumen’, and ‘rRNA metabolic process’ categories (Figure 5A) (Table S2 in File S1) [15]. These data confirmed that loss of *nom1* indeed affected ribosome-related gene expression.

### Ribosome biogenesis and pre-mRNA splicing are affected by *dg5^nom1^* mutation

**Figure 6:**
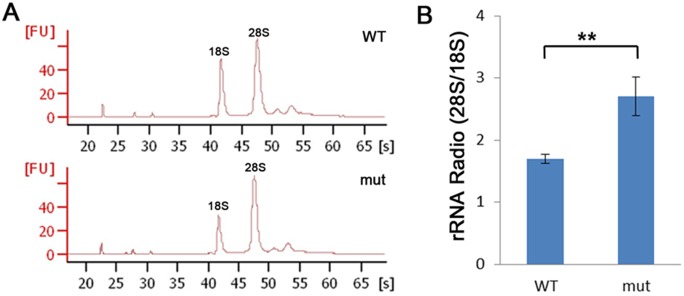
*dg5^nom1^* larvae display defects in ribosome biogenesis. (A) Bioanalyser analysis of total RNA isolated from WT and mutant group at 3 dpf reveals a reduction of the 18S rRNA production but relative normal 28S rRNA amount. (B) The relative rRNA ratio (28S/18S) is elevated in the *dg5^nom1^* as compared to WT. Error bars mean±SD. **P<0.01.

A large pre-rRNA transcript is enzymatically cleaved by ribonucleoprotein complexes in the nucleolus to produces the mature 28S, 18S and 5.8S rRNA [16]. GSEA analysis of the RNA-Seq data revealed that ribosome-related gene expression was affected by *nom1* deficiency, which prompted us to further investigate rRNA processing in *dg5^nom1^* embryos. We found that the production of 18S rRNA was substantially decreased while the 28S rRNA appeared unaffected in *dg5^nom1^* mutant, changing the 28S/18S ratio from 1.7 in WT to 2.7 in *dg5^nom1^* embryos (Figure 6 A, B).

**Figure 7:**
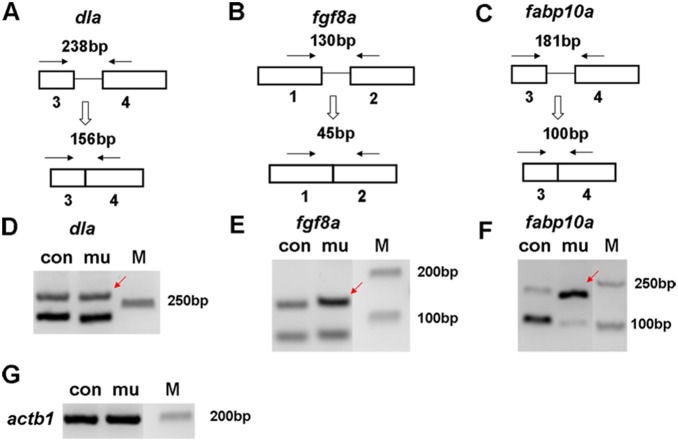
Pre-mRNA splicing of selected genes in *dg5* mutant. (A–C) Schematic illustration of pre-mRNA splicing analysis of *dla*, *fgf8* and *fabp10a* (arrows: primers; boxes: exons; lines: introns). (D–F) Splicing status of *dla*, *fgf8* and *fabp10a* by RT-PCR using primers indicated in A–C. (G) *actb1* is a loading control in each group.

Alexandrov *et al* previously reported that human EIF4AIII had a direct physical interaction with NOM1 [10]. As an essential component of exon junction complex (EJC), EIF4AIII can bind to the upstream DNA sequences of splice junctions [17]. To determine if Nom1 deficiency has an effect on pre-mRNA splicing, we performed bioinformatics analysis of the RNA-Seq data from mutant and wild type control. The Tophat2/Cufflink software was used to detect RNA splice variants through analyzing different exon-exon junctions and their counts from RNA-seq reads, and then assembling the junctions and reads into different transcript forms [18], [19]. This analysis indeed revealed that the pre-mRNA splicing of many genes was affected (Table S3 in File S1). We further selected three genes (*dla*, *fgf8a* and *fabp10a*) and examined the splice forms of them through RT-PCR. The results showed that *fgf8a* and *fabp10a* had more pre-mRNA that remained unspliced in the *dg5^nom1^* mutants but the splicing status of *dla* was not affected (Figure 7). Interestingly, *fabp10a* is expressed in the digestive organs and *fgf8a* is expressed in the brain of zebrafish, two organs that are affected in *dg5^nom1^* mutants. These results suggest that the process of pre-mRNA splicing may contribute specifically to organ defects observed in *dg5^nom1^* mutant.

### Loss of p53 does not rescue pancreas defect in *dg5^nom1^* mutant

**Figure 8:**
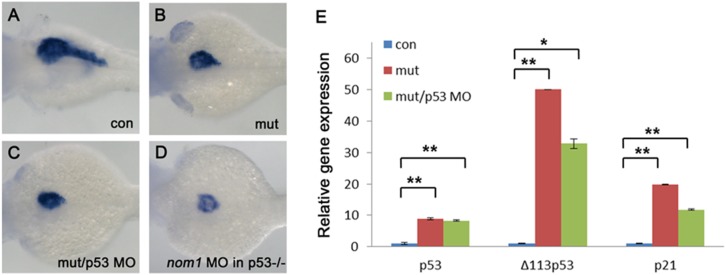
*Nom1*-induced pancreas defect is p53 independent. (A–D) Lateral views, anterior to the left. (A) control group at 3 dpf. (B) *dg5* mutant group at 3 dpf. (C) Failure to rescue the pancreas defect by injection of 4 ng p53MO into *dg5* mutant or (D) injection of *nom1* MO in p53*^M214K^* mutant. (E) Expression of p53 and its targets *Δ1113p53* and *p21* are increased in *dg5^nom1^* mutant, as assessed by quantitative PCR. Error bars mean±SD. **P<0.01, *P<0.05.

Previous works suggest that a p53-dependent mechanism might mediate defective phenotypes associated with ribosome biogenesis [20]–[23]. To determine whether *dg5^nom1^* phenotype was p53-dependent, we first evaluated p53 and its target genes *Δ113p53* and *p21* expression level by qRT-PCR. Compared to control group, p53, *Δ113p53,* and *p21* expression level were all increased in *dg5^nom1^* mutant (Figure 8E). Knockdown of p53 activation by injecting 4 ng of a p53^spl^ MO into *dg5^nom1^* mutant reduced expression level of p53 targets (Figure 8E), but the pancreas defect was not rescued (Figure 8A–C). In order to exclude that the phenotype was due to residual p53 activity, a p53 null mutant line *(tp53^M214K^)* was used. Again, no rescue was observed for the pancreatic phenotype after injection of *nom1*-MO into *p53^−/−^* mutant embryos (Figure 8D), suggesting that the lack of *Nom1* induced a pancreatic defect that is independent of p53.

### Nucleolar *Pp1α* partially rescues the pancreas defect in *nom1* morphant

**Figure 9:**
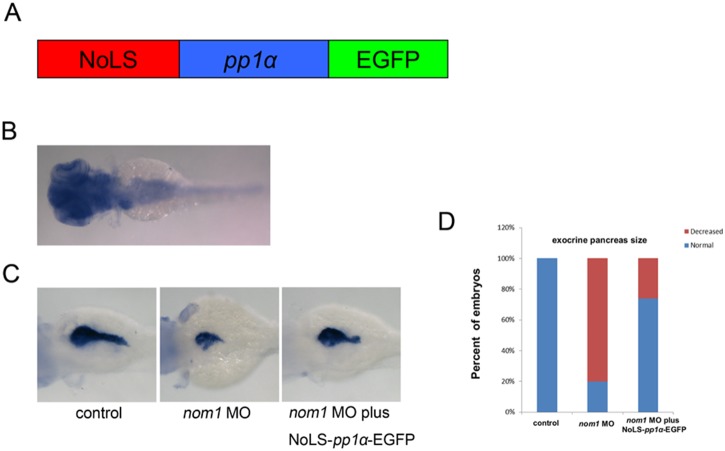
NoLS-*pp1α*-EGFP mRNA partially rescues the pancreas defect in *nom1* morphant. (A) Schematic diagram of NoLS-*pp1α*-EGFP. Red: human *nom1* NoLS. Blue: *pp1α* coding sequence. Green: EGFP coding sequence. (B) *In situ* result demonstrates that *pp1α* expresses in brain, liver, pancreas and intestine at 3 dpf. (C) Co-injection of NoLS-*pp1α*-EGFP mRNA and *nom1* MO partially restores *try* expression as compared to *nom1* morphant. (D) The percentage of embryos with relatively normal exocrine pancreas size is statistically higher in *nom1* MO and NoLS-*pp1α*-EGFP co-injection group.

Protein phosphatase I (Pp1) is a serine/threonine phosphatase that is required for regulating cell cycle, cell signaling, as well as other cellular processes [24], [25]. Gunawardena *et al* reported that NOM1 can target various protein phosphatases to the nucleolus, including PP1α. This process depends on a NOM1 NoLS that is required for nucleolar localization [11]. *In situ* for *pp1α* showed that it is also expressed in liver, pancreas, and intestine (Figure 9B). Additionally, overexpression of *pp1α* mRNA could not rescue the defect in *nom1* morphant. According to the previous work, co-transfection of NOM1-(1-350NoLS)-mCherry and PP1α-eGFP plasmids could lead to a dramatic accumulation of PP1α-eGFP protein in the nucleoli, suggesting that the NoLS of human NOM1 can target PP1α into the nucleoli [11]. To investigate if incorrect distribution of Pp1α is involved in the *dg5^nom1^* mutant phenotype, we cloned the human *nom1* NoLS and artificially fused it to *pp1α* (NoLS-*pp1α*-EGFP) (Figure 9A). We found that injecting the NoLS-*pp1α*-EGFP mRNA could indeed partially restore *try* expression in *nom1* morphants (Figure 9C, 9D, N = 60, ∼50% partial rescue). This suggests that *nom1*-mediated subcellular location of Pp1α plays a key role in controlling pancreas expansion during development.

## Discussion

In this study, we identified a zebrafish mutant (*dg5)* and functionally characterized the mutated gene to be *nom1*, which has been previously shown to regulate pre-rRNA splicing in various organism. In zebrafish, *nom1* mutation results in a decreased level of mature 18S rRNA production. Thus, Nom1 plays conserved roles in the pre-rRNA processing. In *dg5^nom1^* zebrafish model, the exocrine pancreas is the most affected organ. Further studies suggest that specification of exocrine pancreas is largely normal but the proliferation rate is markedly reduced. Homozygous embryos died by 10 dpf, which demonstrates that Nom1 protein is indispensable for larval survival.

Ribosome biogenesis genes, such as nucleolar genes, are generally considered “housekeeping genes” playing general roles in cellular function. However, growing evidence suggests that these genes have tissue specific functions as well, as seen with RNA polymerase III [26], ribosome biogenesis factor Wdr43 [27] and nucleolar protein RBM19 [28]. When these genes are mutated, the zebrafish show defects in specific organs during development. Although *nom1* is essential for zebrafish larval survival, the *dg5^nom1^* embryos appear indistinguishable compared to WT and heterozygous siblings prior to 2.5 dpf. The predominant tissue-restricted phenotypes in *dg5^nom1^* can be contributed to the enriched expression of *nom1*, and presumably function, in pancreas, intestine, and liver.

Gene ontology analysis showed that ribosome-related gene expression was greatly affected by *nom1* deficiency. Further studies demonstrate that production of 18S rRNA was recognizably decreased in *dg5^nom1^* mutant. It is generally accepted that disruption of ribosome biogenesis causes nucleolar stress, and defects in 18S rRNA processing can activate p53 [29]. Quantitative PCR results showed that in *dg5^nom1^* larvae, p53 itself and its downstream genes expression were markedly increased. In some cases, phenotypes caused by ribosome biogenesis defect can be rescued by inhibition of p53 expression [23], [30], [31] but not in other [32]–[34]. In *dg5^nom1^* mutant, neither the p53 MO nor the p53 null mutation could rescue the pancreatic defect, suggesting that p53 independent pathways are involved.

There are three major isoforms of PP1 catalytic subunit (PP1α, PP1β, and PP1γ) in vertebrates. Distinct populations of PP1 are dynamically targeted to different subcellular locations [35]. Gunawardena *et al* reported that NOM1 could target PP1α to the nucleolus through a NoLS. Overexpression of NOM1 leads to accumulation of EGFP-tagged PP1α in nucleoli [11]. In our model, we found that injection of NoLS-*pp1α*-EGFP mRNA could indeed partially restore *try* expression in *nom1* morphants, demonstrating that distribution of PP1 catalytic subunit is important for pancreas development in zebrafish.

Recently, Boglev et al reported that autophagy is a survival mechanism involved in ribosomal stress [32]. In the *tti^s450^* mutant, which has a similar phenotype to our mutant, autophagy is up-regulated. Further studies demonstrated that autophagy induction is independent of Tor pathway and p53. Since ribosomal stress is induced in our *dg5^nom1^* mutant, we believe that autophagy is likely involved in our model. Further investigations of the relationship between the *nom1* function and autophagy should lead to a better understanding of the mechanism.

EIF4AIII is a member of DExD/H-box RNA helicase family, which has been shown to play important roles in all aspects of RNA metabolism, including pre-mRNA splicing, rRNA biogenesis, transcription and RNA stability [36]. Alexandrov *et al* reported that human EIF4AIII has a direct physical interaction with NOM1 [10]. Analysis of RNA-Seq data from *dg5^nom1^* mutant showed that splicing of many genes was affected, including transcripts for *fgf8a* and *fabp10a* mRNA. There have been several reports showing that RNA splicing-related factors have tissue-specific function during vertebrate development such as *usp39* [37], *p110* [38] and *sfpq* [39]. Defects in pre-mRNA splicing process can affect pancreas and brain development, as evidenced by studies on Ddx46, also a member of DExD/H-box proteins [40]. It will be interesting to determine if some of the affected mRNA transcripts contribute to the pancreas phenotype of *dg5^nom1^* mutant.

## Materials and Methods

### Zebrafish husbandry

Wild type TU fish line, *tp53^M214K^* [41], the transgenic line *ptf1a:eGFP*, *kdrl:GFP* (obtained from ZIRC, Eugene, OR) were raised and maintained in a re-circulating aquaculture system according to standards described in The Zebrafish Book [42]. Embryos were incubated at 28.5°C and staged according to the description by Kimmel et al [43]. Heterozygous mutants were crossed to WIK fish lines to generate a mapping population. All animal experiments were approved by Institutional Animal Care and Use Committee (IACUC) of Peking University. The reference from IACUC of Peking University is LSC-ZhangB-1.

### Genetic Mapping and Positional Cloning of *dg5* Locus

Positional cloning of *dg5* locus was performed as described [44]. Two linked markers, Z21519 and G45123 were first identified. Subsequent analysis identified other closely positioned markers, G39065 (4 recombinants in 854 meiosis events), Z1059 marker (14 recombinants in 854 meiosis events) and G40086 (0 recombinant in 854 meiosis events).

### Genotyping and morpholino, mRNA synthesis and microinjection

Homozygous *dg5* larvae were identified via PCR amplification. The primers used were: 5′-GCAGAAGACTAAAAAAGGCG-3′, 5′-TACCTCCTCATCATCTATTT-3′. Two MOs were designed and purchased from Gene Tools Inc (Philomath, OR). The sequence of *nom1* ATG-MO is 5′-GCGCTGCCGCTTTGCCTTCATTTTC-3′, and splicing MO is 5′-AACTGAAGATCAAATACCTCCAGGC-3′, which targeted the boundary of intron 3–4 and exon4. The sequence of p53 MO was described by Langheinrich et al [45]. One or two cell stage embryo was injected at 6 ng for *nom1* ATG MO and 16 ng for *nom1* splicing MO. For validation of the ATG-MO, an EGFP fusion plasmid with *nom1* ATG-MO target site was generated. The primers are: 5′-GGATCCGAAAATGAAGGCAAAGCGGCAGCGCGTGAGCAAG-3′, 5′-CTCGAGTTACTTGTACAGCTCGTCCATGCCGAGAGTGATC-3′. The *nom1* overexpression construct was generated by subcloning full-length *nom1* cDNA from vector pMD19-T simple into vector pCS2+. Primers are 5′-GGATCCATGAAGGCAAAGCGGCAGCG-3′, 5′-CTCGAGCTATAGCTTGATTTTAGCGT-3′. The pCS2+-*nom1* vector was linearized with *kpnI* and mRNA was transcribed using SP6 mMessage mMachine kit (Ambion). For construction of NoLS(h)-*pp1α*-EGFP plasmid, three PCR fragments were generated, and subcloned into pCS2+ vector.

### Whole-mount *in situ* hybridization

Antisense RNA probes were labeled with digoxigenin (DIG) and transcribed by T7/SP6 RNA polymerase (Promega, Madison, WI). WISH was performed as described with NBT/BCIP (Roche) as substrate. The following probes were used: *nom1. ceruloplasmin, hhex, prox1, gata6, insulin, trypsin, ifabp,pp1a, mpx,hbae1,glu,sst.* Larvae were imaged under a fluorescent microscope. (Zeiss, Oberknochen, Germany).

### Analysis of RNA processing

Total RNA from WT and *dg5* mutant was isolated at 3 dpf using mMESSAGE mMACHINE kit (Ambion, Austin, TX) and purified by RNeasy mini kit (Qiagen, Hilden, Germany). Then RNA was analyzed on an Agilent 2100 E-Bioanalyser according to the manufacture’s instruction.

### Immunocytochemistry and TUNEL assay

Whole-mount antibody staining was performed using a rabbit anti phospho-histone H3 (pH3) primary antibody (Cell Signaling Technology) at 1∶100 dilution. Mouse anti-GFP antibody (1∶1000) (Proteintech) was used to detect GFP in *Ptf1a*: GFP transgenic line. A Cy3 labeled goat anti-rabbit second antibody (1∶200) (Proteintech) and an Alexa Fluor 488 conjugated goat anti-mouse second antibody (1∶200) (Invitrogen) were used as secondary antibodies. For TUNEL assay, transverse sections were prepared using a Leica VT1000S vibratome at 200 µm intercals. Then the staining was performed using In Situ Cell Death Detection Kit (Roche) according to the manufacture’s instructions. The images were then observed under a Zeiss LSM 510 meta confocal microscope (Zeiss, Oberkochen, Germany).

### Alcian blue staining

Alcian blue (Sigma) was used for skeletal staining of 4 dpf (days post-fertilization) embryos as described previously [46].

### RNA-Seq analysis

Whole RNA from Wild type and mutant embryos at 2.5 dpf were sequenced using the Illumina HiSeq 2000 system. All reads were mapped to the zebrafish genome (version Zv9) and exon-exon juntions were detected using Tophat [18]. All mapped reads were then assigned and assembled into different transcript isforms using Cufflinks [19]. Differential expression and splicing were calculated using Cuffdiff [14]. Genes with expression fold change ≥2 and q-value<0.05 were determined as significantly differentially expressed. The same q-value threshold was used for differential-splicing analysis. Gene set enrichment analysis was performed using GSEA, according to the GO functional categories from MSigDB [47]. GO terms were considered as significantly enriched (or depleted) if the q-value<0.05.

### RT-PCR and Quantitative RT-PCR

cDNA was synthesized using Primer Script RT reagent Kit (Takala). Real time RT-PCR primers were used as described [48]. Gene expression was quantified using 7300 Real time PCR system.

### Statistical methods

Experiments were independently repeated at least three times. Then Mean and SEM were calculated. P values were calculated using a two-sided unpaired Student’s t-test and less than 0.05 was considered as significant.

## Supporting Information

File S1 **This file contains Figure S1-Figure S5 and Table S1-Table S3. Figure S1,** Endocrine pancreas markers expression was not affected in *dg5* mutant larvae. (A, B) the endocrine pancreas β-cell marker *ins* was not affected in *dg5* mutant at 3 dpf. (C–F) The same result can be seen in pancreas α-cell markern *glu* and pancreas δ–cell marker *sst*. All dorsal views, anterior to the left. **Figure S2,** *Dg5* larvae does not have an effect on hematopoiesis. (A–D) *dg5* has a normal expression pattern on hemoglobin marker *hbae1* and myeloid marker *mpo* compared to WT group. All lateral views, anterior to the left. **Figure S3,** The efficiency verification of *nom1* ATG MO. (A) Strong GFP fluorescence appeared in embryos injected with a fusion EGFP protein contained *nom1* ATG MO target site. (B) The GFP fluorescence is disappeared in embryos co-injected with the fusion protein mRNA and 2 ng *nom1* ATG MO. **Figure S4,** Phenotype of *dg5* larvae at 7 dpf and the morphology of *nom1*-knockdown morphant and rescue embryos. (A, B) *dg5* larvae do not have a swim bladder and cause a serious edema (arrow) at 7 dpf. (C) Compared to control embryos, (D) *nom1* ATG-MO can cause the same phenotype as *dg5* mutant with small head, small eyes and heart edema. (E) Injection *nom1* mRNA into *dg5* mutant can rescue the morphology phenotype. All lateral views, anterior to the left. **Figure S5,** *Nom1* affect exocrine pancreas development process between 2 dpf and 2.5 dpf. (A–F) All dorsal views, anterior to the top. (A, D) In morphant group, the GFP labeled exocrine pancreas size is the same as WT. (B, C, E, F) The exocrine pancreas began to enlarge in WT larvae, but not for the *nom1*-knockdown embryos at 2.5 dpf and 3 dpf. **Table S1,** Differentially expressed genes in *dg5* versus sibilings at 2.5 dpf. **Table S2,** GO enrichment analysis in *dg5* mutant. **Table S3,** Isoforms analysis in *dg5* mutant.
(ZIP)
