# Supplementary figures and images for: Nom1 Mediates Pancreas Development by Regulating Ribosome Biogenesis in Zebrafish
Source: PLoS One. 2014 Jun 26;9(6):e100796. doi: 10.1371/journal.pone.0100796 (PMC4072693; doi:10.1371/journal.pone.0100796)

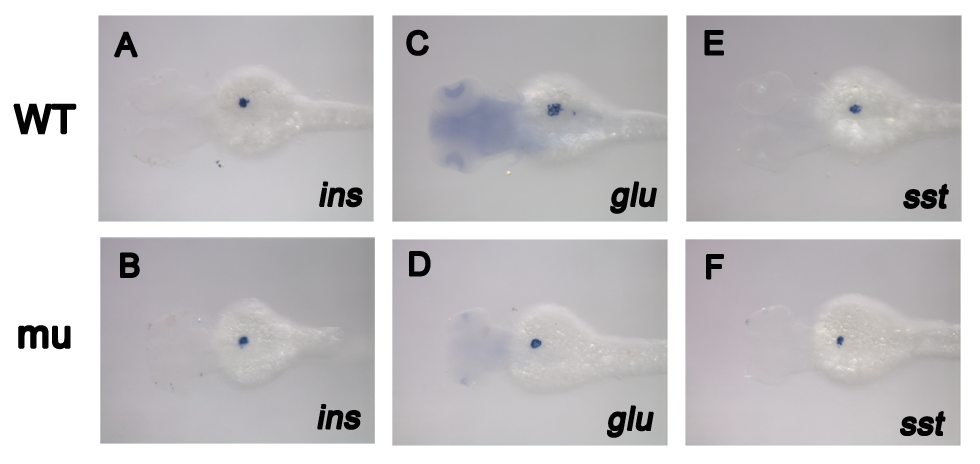

Supplement: File S1 — This file contains Figure S1-Figure S5 and Table S1-Table S3. Figure S1, Endocrine pancreas markers expression was not affected in dg5 mutant larvae. (A, B) the endocrine pancreas β-cell marker ins was not affected in dg5 mutant at 3 dpf. (C–F) The same result can be seen in pancreas α-cell markern glu and pancreas δ–cell marker sst. All dorsal views, anterior to the left. Figure S2, Dg5 larvae does not have an effect on hematopoiesis. (A–D) dg5 has a normal expression pattern on hemoglobin marker hbae1 and myeloid marker mpo compared to WT group. All lateral views, anterior to the left. Figure S3, The efficiency verification of nom1 ATG MO. (A) Strong GFP fluorescence appeared in embryos injected with a fusion EGFP protein contained nom1 ATG MO target site. (B) The GFP fluorescence is disappeared in embryos co-injected with the fusion protein mRNA and 2 ng nom1 ATG MO. Figure S4, Phenotype of dg5 larvae at 7 dpf and the morphology of nom1-knockdown morphant and rescue embryos. (A, B) dg5 larvae do not have a swim bladder and cause a serious edema (arrow) at 7 dpf. (C) Compared to control embryos, (D) nom1 ATG-MO can cause the same phenotype as dg5 mutant with small head, small eyes and heart edema. (E) Injection nom1 mRNA into dg5 mutant can rescue the morphology phenotype. All lateral views, anterior to the left. Figure S5, Nom1 affect exocrine pancreas development process between 2 dpf and 2.5 dpf. (A–F) All dorsal views, anterior to the top. (A, D) In morphant group, the GFP labeled exocrine pancreas size is the same as WT. (B, C, E, F) The exocrine pancreas began to enlarge in WT larvae, but not for the nom1-knockdown embryos at 2.5 dpf and 3 dpf. Table S1, Differentially expressed genes in dg5 versus sibilings at 2.5 dpf. Table S2, GO enrichment analysis in dg5 mutant. Table S3, Isoforms analysis in dg5 mutant. (ZIP) [file pone.0100796.s001.zip › File S1/Figure S1.tif]

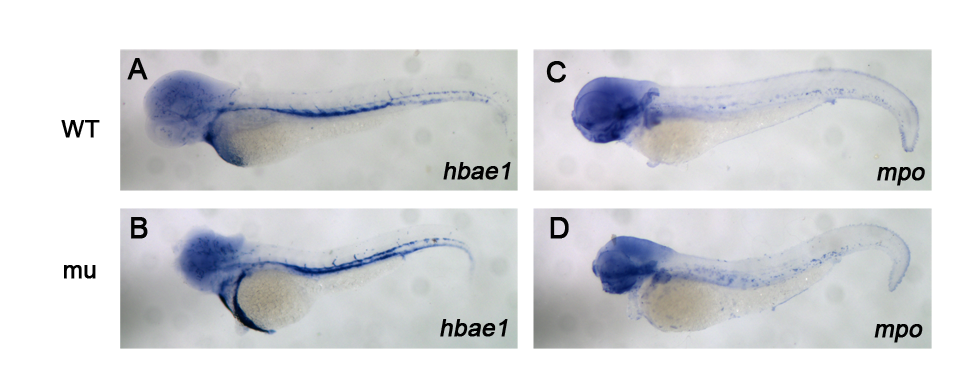

Supplement: File S1 — This file contains Figure S1-Figure S5 and Table S1-Table S3. Figure S1, Endocrine pancreas markers expression was not affected in dg5 mutant larvae. (A, B) the endocrine pancreas β-cell marker ins was not affected in dg5 mutant at 3 dpf. (C–F) The same result can be seen in pancreas α-cell markern glu and pancreas δ–cell marker sst. All dorsal views, anterior to the left. Figure S2, Dg5 larvae does not have an effect on hematopoiesis. (A–D) dg5 has a normal expression pattern on hemoglobin marker hbae1 and myeloid marker mpo compared to WT group. All lateral views, anterior to the left. Figure S3, The efficiency verification of nom1 ATG MO. (A) Strong GFP fluorescence appeared in embryos injected with a fusion EGFP protein contained nom1 ATG MO target site. (B) The GFP fluorescence is disappeared in embryos co-injected with the fusion protein mRNA and 2 ng nom1 ATG MO. Figure S4, Phenotype of dg5 larvae at 7 dpf and the morphology of nom1-knockdown morphant and rescue embryos. (A, B) dg5 larvae do not have a swim bladder and cause a serious edema (arrow) at 7 dpf. (C) Compared to control embryos, (D) nom1 ATG-MO can cause the same phenotype as dg5 mutant with small head, small eyes and heart edema. (E) Injection nom1 mRNA into dg5 mutant can rescue the morphology phenotype. All lateral views, anterior to the left. Figure S5, Nom1 affect exocrine pancreas development process between 2 dpf and 2.5 dpf. (A–F) All dorsal views, anterior to the top. (A, D) In morphant group, the GFP labeled exocrine pancreas size is the same as WT. (B, C, E, F) The exocrine pancreas began to enlarge in WT larvae, but not for the nom1-knockdown embryos at 2.5 dpf and 3 dpf. Table S1, Differentially expressed genes in dg5 versus sibilings at 2.5 dpf. Table S2, GO enrichment analysis in dg5 mutant. Table S3, Isoforms analysis in dg5 mutant. (ZIP) [file pone.0100796.s001.zip › File S1/Figure S2.tif]

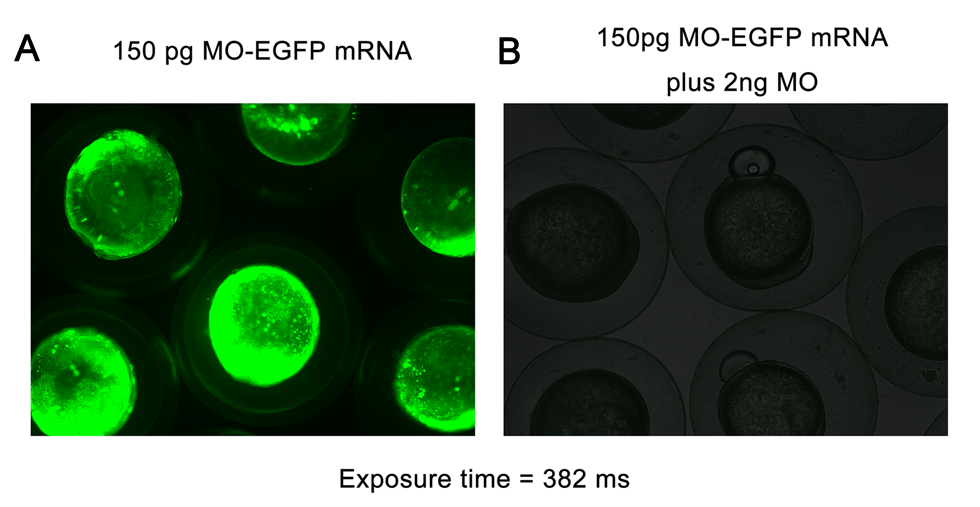

Supplement: File S1 — This file contains Figure S1-Figure S5 and Table S1-Table S3. Figure S1, Endocrine pancreas markers expression was not affected in dg5 mutant larvae. (A, B) the endocrine pancreas β-cell marker ins was not affected in dg5 mutant at 3 dpf. (C–F) The same result can be seen in pancreas α-cell markern glu and pancreas δ–cell marker sst. All dorsal views, anterior to the left. Figure S2, Dg5 larvae does not have an effect on hematopoiesis. (A–D) dg5 has a normal expression pattern on hemoglobin marker hbae1 and myeloid marker mpo compared to WT group. All lateral views, anterior to the left. Figure S3, The efficiency verification of nom1 ATG MO. (A) Strong GFP fluorescence appeared in embryos injected with a fusion EGFP protein contained nom1 ATG MO target site. (B) The GFP fluorescence is disappeared in embryos co-injected with the fusion protein mRNA and 2 ng nom1 ATG MO. Figure S4, Phenotype of dg5 larvae at 7 dpf and the morphology of nom1-knockdown morphant and rescue embryos. (A, B) dg5 larvae do not have a swim bladder and cause a serious edema (arrow) at 7 dpf. (C) Compared to control embryos, (D) nom1 ATG-MO can cause the same phenotype as dg5 mutant with small head, small eyes and heart edema. (E) Injection nom1 mRNA into dg5 mutant can rescue the morphology phenotype. All lateral views, anterior to the left. Figure S5, Nom1 affect exocrine pancreas development process between 2 dpf and 2.5 dpf. (A–F) All dorsal views, anterior to the top. (A, D) In morphant group, the GFP labeled exocrine pancreas size is the same as WT. (B, C, E, F) The exocrine pancreas began to enlarge in WT larvae, but not for the nom1-knockdown embryos at 2.5 dpf and 3 dpf. Table S1, Differentially expressed genes in dg5 versus sibilings at 2.5 dpf. Table S2, GO enrichment analysis in dg5 mutant. Table S3, Isoforms analysis in dg5 mutant. (ZIP) [file pone.0100796.s001.zip › File S1/Figure S3.tif]

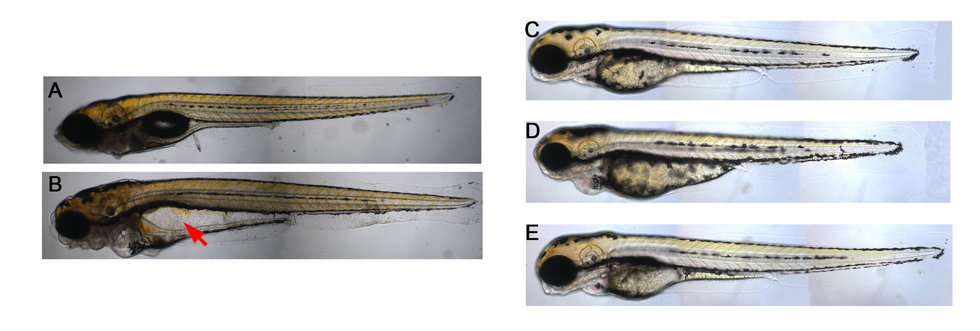

Supplement: File S1 — This file contains Figure S1-Figure S5 and Table S1-Table S3. Figure S1, Endocrine pancreas markers expression was not affected in dg5 mutant larvae. (A, B) the endocrine pancreas β-cell marker ins was not affected in dg5 mutant at 3 dpf. (C–F) The same result can be seen in pancreas α-cell markern glu and pancreas δ–cell marker sst. All dorsal views, anterior to the left. Figure S2, Dg5 larvae does not have an effect on hematopoiesis. (A–D) dg5 has a normal expression pattern on hemoglobin marker hbae1 and myeloid marker mpo compared to WT group. All lateral views, anterior to the left. Figure S3, The efficiency verification of nom1 ATG MO. (A) Strong GFP fluorescence appeared in embryos injected with a fusion EGFP protein contained nom1 ATG MO target site. (B) The GFP fluorescence is disappeared in embryos co-injected with the fusion protein mRNA and 2 ng nom1 ATG MO. Figure S4, Phenotype of dg5 larvae at 7 dpf and the morphology of nom1-knockdown morphant and rescue embryos. (A, B) dg5 larvae do not have a swim bladder and cause a serious edema (arrow) at 7 dpf. (C) Compared to control embryos, (D) nom1 ATG-MO can cause the same phenotype as dg5 mutant with small head, small eyes and heart edema. (E) Injection nom1 mRNA into dg5 mutant can rescue the morphology phenotype. All lateral views, anterior to the left. Figure S5, Nom1 affect exocrine pancreas development process between 2 dpf and 2.5 dpf. (A–F) All dorsal views, anterior to the top. (A, D) In morphant group, the GFP labeled exocrine pancreas size is the same as WT. (B, C, E, F) The exocrine pancreas began to enlarge in WT larvae, but not for the nom1-knockdown embryos at 2.5 dpf and 3 dpf. Table S1, Differentially expressed genes in dg5 versus sibilings at 2.5 dpf. Table S2, GO enrichment analysis in dg5 mutant. Table S3, Isoforms analysis in dg5 mutant. (ZIP) [file pone.0100796.s001.zip › File S1/Figure S4.tif]

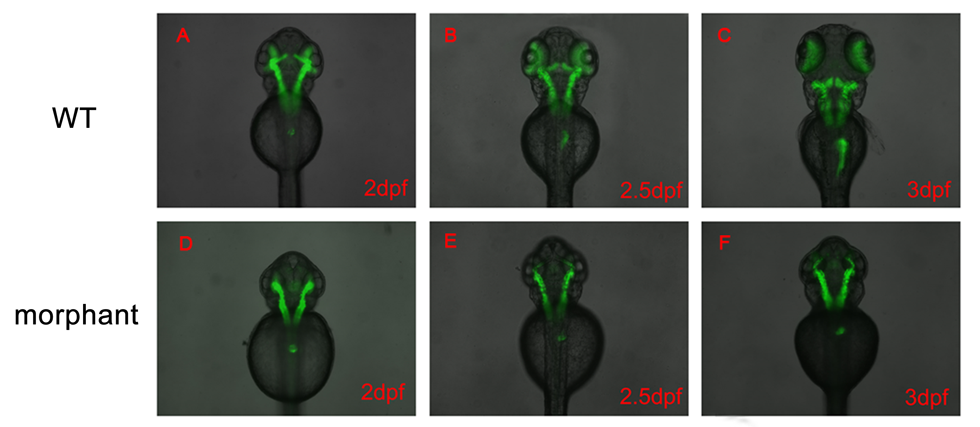

Supplement: File S1 — This file contains Figure S1-Figure S5 and Table S1-Table S3. Figure S1, Endocrine pancreas markers expression was not affected in dg5 mutant larvae. (A, B) the endocrine pancreas β-cell marker ins was not affected in dg5 mutant at 3 dpf. (C–F) The same result can be seen in pancreas α-cell markern glu and pancreas δ–cell marker sst. All dorsal views, anterior to the left. Figure S2, Dg5 larvae does not have an effect on hematopoiesis. (A–D) dg5 has a normal expression pattern on hemoglobin marker hbae1 and myeloid marker mpo compared to WT group. All lateral views, anterior to the left. Figure S3, The efficiency verification of nom1 ATG MO. (A) Strong GFP fluorescence appeared in embryos injected with a fusion EGFP protein contained nom1 ATG MO target site. (B) The GFP fluorescence is disappeared in embryos co-injected with the fusion protein mRNA and 2 ng nom1 ATG MO. Figure S4, Phenotype of dg5 larvae at 7 dpf and the morphology of nom1-knockdown morphant and rescue embryos. (A, B) dg5 larvae do not have a swim bladder and cause a serious edema (arrow) at 7 dpf. (C) Compared to control embryos, (D) nom1 ATG-MO can cause the same phenotype as dg5 mutant with small head, small eyes and heart edema. (E) Injection nom1 mRNA into dg5 mutant can rescue the morphology phenotype. All lateral views, anterior to the left. Figure S5, Nom1 affect exocrine pancreas development process between 2 dpf and 2.5 dpf. (A–F) All dorsal views, anterior to the top. (A, D) In morphant group, the GFP labeled exocrine pancreas size is the same as WT. (B, C, E, F) The exocrine pancreas began to enlarge in WT larvae, but not for the nom1-knockdown embryos at 2.5 dpf and 3 dpf. Table S1, Differentially expressed genes in dg5 versus sibilings at 2.5 dpf. Table S2, GO enrichment analysis in dg5 mutant. Table S3, Isoforms analysis in dg5 mutant. (ZIP) [file pone.0100796.s001.zip › File S1/Figure S5.tif]
